# Evaluation of Deoxyribonucleic Acid Toxicity Induced by the Radiopharmaceutical ^99m^Technetium-Methylenediphosphonic Acid and by Stannous Chloride in Wistar Rats 

**DOI:** 10.3390/molecules171112974

**Published:** 2012-11-01

**Authors:** José Carlos Pelielo De Mattos, Vanessa Coutinho de Matos, Michelle Pinheiro Rodrigues, Marcia Betânia Nunes de Oliveira, Flavio José S. Dantas, Sebastião David Santos-Filho, Mario Bernardo-Filho, Adriano Caldeira-de-Araujo

**Affiliations:** Departamento de Biofísica e Biometria, Instituto de Biologia Roberto Alcantara Gomes, Universidade do Estado do Rio de Janeiro, Av. 28 de Setembro, 87, Rio de Janeiro 20551-030, Brazil; Email: jcmattos@uerj.br (J.C.P.D.M.); vanessacmatos@gmail.com (V.C.M.); michellerodr@gmail.com (M.P.R.); mbno@ig.com.br (M.B.N.O.); flaviojsdantas@gmail.com (F.J.S.D.); sdavidsfilho@gmail.com (S.D.S.-F.); caldeiradearaujo@gmail.com (A.C.-A.)

**Keywords:** stannous chloride, ^99m^Tc-MDP, genotoxicity, mutagenicity, Comet assay, micronucleus test

## Abstract

Radiopharmaceuticals are employed in patient diagnostics and disease treatments. Concerning the diagnosis aspect, technetium-99m (^99m^Tc) is utilized to label radiopharmaceuticals for single photon computed emission tomography (SPECT) due to its physical and chemical characteristics. ^99m^Tc fixation on pharmaceuticals depends on a reducing agent, stannous chloride (SnCl_2_) being the most widely-utilized. The genotoxic, clastogenic and anegenic properties of the ^99m^Tc-MDP(methylene diphosphonate used for bone SPECT) and SnCl_2_ were evaluated in Wistar rat blood cells using the Comet assay and micronucleus test. The experimental approach was to endovenously administer NaCl 0.9% (negative control), cyclophosphamide 50 mg/kg b.w. (positive control), SnCl_2_ 500 μg/mL or ^99m^Tc-MDP to animals and blood samples taken immediately before the injection, 3, and 24 h after (in the Comet assay) and 36 h after, for micronucleus test. The data showed that both SnCl_2_ and ^99m^Tc-MDP-induced deoxyribonucleic acid (DNA) strand breaks in rat total blood cells, suggesting genotoxic potential. The ^99m^Tc-MDP was not able to induce a significant DNA strand breaks increase in *in vivo* assays. Taken together, the data presented here points to the formation of a complex between SnCl_2_ in the radiopharmaceutical ^99m^Tc-MDP, responsible for the decrease in cell damage, compared to both isolated chemical agents. These findings are important for the practice of nuclear medicine.

## 1. Introduction

Radiopharmaceuticals are widely employed in scintigraph examinations to aid in diagnosis or to treat diseases. This use involves various health and environmental risks, as many of the side effects of these compounds are not fully understood. Concerning diagnosis, the most used radionuclide to perform single photon emission computed tomography (SPECT) is technetium-99m (^99m^Tc) due to its useful physical and chemical characteristics [1,2].

^99m^Tc is obtained from a ^99^Mo/^99m^Tc generator in the form of sodium pertechnetate (Na^99m^TcO_4_), which displays low chemical reactivity, being this the reason why the use of a reducing agent for binding the radionuclide to the pharmaceutical becomes necessary. To date, the most efficient reducing agent for ^99m^Tc has been stannous chloride (SnCl_2_), which renders it the most frequently used compound for this purpose [2,3,4].

Besides being used in the scintigraph examinations in nuclear medicine, stannous ion is also used for other purposes. For instance, it is used for metallic food packages [5,6,7], as a fluoride vehicle in toothpastes and mouthwashes [8,9], as a soft drink conservant [10]. Tin organometallic compounds are used as stabilizers in perfumes, soaps and PVC [11], as well as in biocides [12] and, combined with other chemical substances, for testing cancer treatments [13].

The toxic effects of stannous salts are still being investigated, although they are known to include the following: irritation of oral mucosa and possible inflammation in rats [14]; severe intoxication from consuming foods or drinks from stannous containers [5]; inducing carcinogenic activity in the thyroid glands of rats [15]; allowing calcium entry into motor nerve terminals in mice [16]; and in osteoblast cultures [17]. Moreover, when administered orally, this agent can change enzymatic activity, renal and hepatic histopathology, as well as cause lipid peroxidation in male rabbits [11]. There is also a decrease of reproductive performance because of low sperm quality in these animals [18]. The absence of mutagenic effects has been reported by Nishioka [19], Kada *et al.* [20] and Singh [21], although this view is contested by Olivier and Marzin [22]. Stannous chloride is capable of causing mutations in the supF gene of pAC 189 vector, as described by Cabral *et al.* [23], who agreed with Pungartnik *et al.* [24] that this salt should be classified as a moderate low toxicity mutagen.

The occurrence of double and single strand breaks in pUC 9.1 plasmid DNA caused by the stannous chloride, which alters the efficiency of transformation of this same plasmid into *E. coli* AB 1157 strain was shown by Dantas *et al.* [25]. 

Stannous chloride’s toxic effects may be due either to an indirect action, mediated by reactive oxygen species (ROS), generated in a Fenton-like reaction [26], or to a direct action, where it is linked to the DNA molecule [27,28]. The side effects of stannous chloride, when used in nuclear medicine kits, are still little understood, although glucoheptonic acid can decrease the harmful effects of this salt in prokaryotic cells [29].

Methylene diphosphonate (MDP) is one of several pharmaceuticals labeled with ^99m^Tc, being used for skeletal SPECT imaging, since it is an analogous organic pyrophosphate, having a strong affinity for bone tissue [2,30].

In experiments carried out with bacterial strains, Guedes *et al.* [31] pointed to a high toxicity level when cells were incubated with separate MDP and SnCl_2_, although a decrease in this toxic effect was observed in samples incubated with both agents together. Thus, the aim of this work was to evaluate the effects induced by ^99m^Tc-MDP and stannous chloride in the blood and bone marrow cells of Wistar rats using two assays related to the molecular biology (Comet Assay and Micronucleus Test).

## 2. Results and Discussion

**Figure 1:**
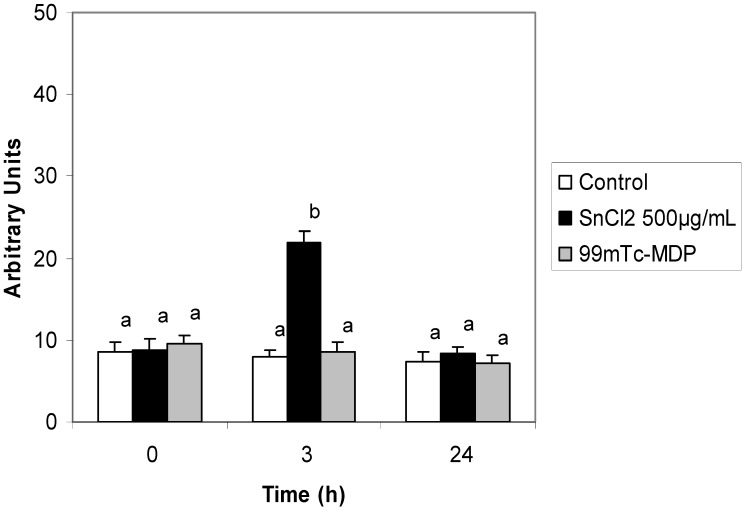
Comet assay of total blood cells of Wistar rats treated with NaCl 0.9% (Control); SnCl_2_ 500 µg/mL and ^99m^Tc-MDP. Blood was collected immediately before intravenous injections and in time intervals of 3 and 24 h after chemical solution administration. Results for each procedure comprise means from three experiments; error bars represent standard error mean. Different letters (a,b) correspond to significant (*p* < 0.05) statistical differences.

Figure 1 shows our Comet assay results. The treatment of animals with NaCl 0.9% sterile solution (control group) was not able to induce a statistically significant increase (*p* > 0.05) in the number of DNA lesions of rat blood cells. Despite the high sensitivity of the technique used, which detects low damage levels, the results show that the condition, under which the rats were submitted during the experiment did not interfere with the parameters analyzed. Besides, when the ^99m^Tc-MDP was administered to the rats, there was no genotoxic effect, as the values were similar to those found in the control group (*p* > 0.05).

Figure 1 also shows that the concentration of SnCl_2_ tested (500 µg/mL), was able to induce a statistically significant increment (*p* < 0.001) in comet numbers in peripheral blood nuclear cells, 3 h after exposure. It can also be inferred that, 24 h after the administration, the number of lesions had already fallen back to values close to those of control.

The results obtained from the rat bone marrow cells after 36 h of administration with ^99m^Tc-MDP and SnCl_2_ (500 µg/mL) are shown in Table 1. Micronucleated bone marrow polychromatic erythrocytes (MNPCE) means (per 1,000 PCE) in rats were 1.8 in the group exposed to ^99m^Tc-MDP treatment while that one from the positive control was 16.2 (*p* < 0.05). Thereby, no clastogenic/aneugenic effect was observed in this treatment. Similarly, the administration of SnCl_2_ (500 µg/mL) did not bring about the formation of micronucleus in Wistar rats bone marrow. In this case, The MNPCE means were 2.2 (*p* < 0.05). Both treatments did not exhibit a significant difference when compared to the negative control group (*p* > 0.05). The frequency of three micronuclei to 1,000 PCE’s is regarded as normal.

**Table 1:** Micronucleus Test in Wistar rats bone marrow.

*Micronucleated polychromatic erythrocytes*					
Treatments	No. of Animals	Individual data ^a^	N	%	Mean ± SD
NaCl 0.9%	5	1.2, 2.0, 1.3, 1.2, 2.1	15	0.15	1.50 ^b^ ± 0.85
Cyclophosphamide 50 mg/kg b.w.	5	18.15, 22.13, 15.17, 20.12, 16.14	162	1.62	16.20 ± 3.12
SnCl_2_ 500 μg/mL	5	2.1, 1.3, 2.3, 2.2, 3.3	22	0.22	2.20 ^b,c^ ± 0.79
^99m^Tc-MDP 100 μCi	5	1.2, 2.1, 2.3, 2.2, 2.1	18	0.18	1.80 ^b,c^ ± 0.63

^a^ Per 1,000 polychromatic erythrocytes per rat; ^b^ Statistically different from the positive control (cyclophosphamide 50 mg/kg b.w.). * *p* < 0.05; ^c^ Not statistically different from the negative control (NaCl 0.9%); * *p* > 0.05.

Related to SnCl_2_ genotoxicity, results in Figure 1 show that this effect was only recorded with a 500 µg/mL concentration. However, there is a decrease in SnCl_2_-induced lesions after 24 h, which could be explained by: (i) an efficient cellular function repair mechanism; (ii) error-prone repair, which could bring about mutagenesis and even carcinogenesis; (iii) cell death occurrence by apoptosis or necrosis. With metal concentration increases in cells inducing protein expression, these proteins, such as metallothioneins, have the role of maintaining homeostasis of these compounds in the organism. Such proteins are usually found in eukaryotic and in some prokaryotic cells, having an important role in chelating toxic metals and, thus, can regulate ROS formation [32], thereby decreasing production of DNA lesions.

The Divalent Cation Transporter 1 (DCT1) protein, also known as Divalent Metal Transporter 1 (DMT1), is an ion metallic transporter with a wide range of activity. This protein is a transporter of Fe^2+^, Zn^2+^, Mn^2+^, Co^2+^, Cd^2+^, Cu^2+^, Ni^2+^ and Pb^2+^ (all divalent ions), as well as of Sn^+2^, and was described in rats by Gunshin *et al*. [33]. Recent results shown that DMT1 can transport metallic ions through the cell intestinal epitethelium membrane [34], as well as being expressed in mice blood cells [35]. Perhaps, activity jointly with these proteins could be responsible for the low toxicity levels in animal experiments with SnCl_2_. The absence of damage effects when rats were treated with SnCl_2_, at a 250 μg/mL concentration, indicates that this detoxification system was capable of taking out some of the excess of tin present. However, the harmful effect verified for SnCl_2_ concentration of 500 μg/mL, indicates saturation of the detoxification system with high tin concentrations.

There was no genotoxicity with ^99m^Tc-MDP (Figure 1), such as when SnCl_2_ was injected singularly, at a 250 μg/mL concentration, which corresponds to that available in the kit used in the SPECT nuclear medicine procedure. These results could indicate that there is some cellular mechanism in the animals preventing the lesions caused by such agents. A possibility is that this protection was imposed by the activity of metal-binding proteins [32,33,34,36] which, when binding with the stannous ion, thus avoid its direct effects, by connecting to DNA [27,28], as much as by its indirect effect, by preventing the Fenton-like reaction from occurring, in which ROS will be generated [26]. 

Besides, it could be explained that, in the ^99m^Tc-MDP, a number of stannous ions are involved with the reduction of sodium pertechnetate and, therefore, there are fewer free ions available to produce lesions as well as to combine with MDP.

Previous results from our laboratory show that the MDP pharmaceutical displayed accentuated genotoxicity and cytotoxicity in prokaryotes and in plasmid DNA [31]. In the plasmid case, despite not having caused the formation of a specific band of single and/or double strand breaks, there was extended DNA smearing, related to the formation of small different-sized fragments caused by DNA breaks. 

No significant increase in the number of micronucleated cells in both segments was observed in the micronucleus test (Table 1). Thus, ^99m^Tc-MDP, besides not displaying a genotoxic effect (Figure 1), does not bear an aneugenic or clastogenic effect, as it was not able to induce the formation of micronucleated cells in animal bone marrow in the first 36 hours following treatment (Table 1). The result obtained as from the SnCl_2_ treatment (500 μg/mL), which displayed genotoxic potential, bringing forth lesions in rat peripheric blood cell DNA, seems to reflect animal DNA repair systems which, 24 h following the administration of stannous chloride, had already been able to repair the lesions produced by this agent, detected through Comet assay, as demonstrated in Figure 1. Therefore, such lesions were not able to trigger micronuclei formation. These data, even if not conclusive, in addition to those results obtained by Guedes *et al*. [31], suggest that isolated kit compounds, such as SnCl_2_, could induce genotoxic damage, but when administered as a whole kit in rats, it seems to be safe. 

## 3. Experimental

### 3.1. Reagents

Stannous chloride (SnCl_2_·2H_2_O), normal agarose, low-melting point agarose (LMPA), ethidium bromide (EtBr), Tris (tris(hydroxymethyl)aminomethane hydrochloride), disodium ethylenediamine tetraacetate (Na_2_EDTA), dimethyl sulfoxide (DMSO) and Triton X-100 were obtained from Sigma (St Louis, MO, USA); NaOH and NaCl from Merck (St. Louis, MO, USA). Phosphate-buffered saline (PBS) was supplied by Invitrogen (São Paulo, Brazil). A commercial ^99m^Tc-radiopharmaceutical kit containing MDP (5.0 mg) and SnCl_2_·2H_2_O (1 mg) was donated by Laboratório de Radiofarmácia, Universidade Federal de Minas Gerais, Brazil. The labeled kit was kindly donated by the Departamento de Radiologia, Universidade Federal do Rio de Janeiro, Brazil. Milli-Q water was used to prepare all solutions employed in this study, as to minimize metal contamination.

### 3.2. Animals

Adult male Wistar rats (3 months of age, body weight 250–300 g) were obtained from the Fundação Instituto Oswaldo Cruz (Rio de Janeiro, Brazil). Animals were housed in polypropylene cages (six rats per cage) with hardwood-chip bedding in an air-conditioned room at 24 ± 1 °C; 55 ± 5% humidity and with a 12 h light/dark cycle. Rats had free access to water and food and were treated in accordance with the *Guide for the Care and Use of Laboratory Animals* (National Research Council, USA). Animals were randomly allocated in nine groups, (each one with six rats), having been administered a single 100 μL intravenous injection of: group 1—NaCl 0.9% (negative control); group 2—cyclophosphamide 50 mg/kg b.w. (positive control) (only for the micronucleus test); group 3—SnCl_2_ 500 µg/mL; and group 4—^99m^Tc-MDP (^99m^Tc—100 μCi; SnCl_2_—12.5 μg/mL; MDP—333 μg/mL). All agents were dissolved in NaCl 0.9% solution. The animals were sacrificed in a CO_2_ chamber thirty-six hours after the treatment, to secure samples for the micronucleus test.

A higher ^99m^Tc activity is used in a typical human nuclear medicine imaging study [2]. Here, a correlation was done on account of rats’ body weight and ascertaining 100 μCi as an ideal activity. MDP concentration (333 μg/mL) is the same as found in nuclear medicine kits; SnCl_2_ concentration (when used alone) was increased in order to amplify its damaging effects, because when 250 µg/mL SnCl_2_ was administered to the rats, there was no significant increase in the number of DNA strand breaks in the blood cells analyzed (*p* > 0.05)—data not shown.

### 3.3. Comet Assay

The *in vivo* alkaline single cell gel electrophoresis assay, the Comet assay, can be used to investigate the genotoxicity of industrial chemicals, biocides, agrichemicals and pharmaceuticals. It can detect double and single-strand breaks, alkali-labile sites expressed as single-strand breaks and single-strand breaks associated with incomplete excision repair [37]. Figure 1 shows representative images defining the different damage classes of comets.

The procedure below was carried out as described by Speit and Hartmann [37] and used elsewhere [38,39]. Blood was collected from the cut tip of the tails of all rats into heparinized eppendorf tubes, immediately before intravenous injections and at 3 and 24 h after chemical solution administration. Following the treatments, 10 μL total blood aliquots from each animal were mixed with 120 μL prewarmed (37 °C) 0.5% LMP agarose, in PBS, and the whole volume put on a microscope slide previously coated with 1.5% normal agarose, in PBS. Ten slides from each animal, for each time interval studied, were prepared as described above, covered with microscope cover slips and kept at 4 °C for 20 min, to accelerate gelling. Following agarose, slides were submerged for 1 h in a freshly-prepared cold lysis solution (2.5 M NaCl, 10 mM Tris, 100 mM EDTA, 10% DMSO, 1% Triton X-100, pH 10.0), in order to remove cytoplasmic and nuclear proteins. Following that, slides were transferred to an electrophoresis chamber containing a high pH (>13) buffer (300 mM NaOH, 1 mM EDTA) and incubated for 20 min, at room temperature, to allow the DNA to unwind. Electrophoresis was performed at 1.0 V/cm, 300 mA for 25 min. Subsequently, slides were washed three times with neutralization buffer (0.4 M Tris-HCl, pH 7.5), fixed in 100% ethanol, washed with ultrapure water (Milli-Q system) and dried at room temperature, overnight. Slides were stained with ethidium bromide (30 µL, 40 µg/mL) and immediately evaluated at 400x magnification, using a fluorescence microscope equipped with a 580 nm excitation filter and a 590 nm barrier filter set, and quantified as described below.

Damages was assigned based on the visual aspect of the comets, considering the DNA migration pattern, and cells were scored, according to tail length, into four classes (0–3). Class 0 means undamaged nucleus, there being no tail formation. In class 1, the nucleus displays a short tail, smaller than nucleus diameter; class 2, tail length is between 1 and 2 times head diameter. Nucleus maximally damaged is allocated to class 3, where tail is two times, or longer, than nucleus diameter. For each slide, 50 nuclei were randomly observed, always from the left to the right side, total scoring (TS) being obtained by multiplying the number of cells in each class (n_x_) by the damage class (Equation 1), being ranged from 0 to 150:

TS = (0 x n_0_) +(1 · n_1_) + (2 · n_2_) + (3 · n_3_) (Equation 1)

Three independent experiments were carried out for each time period throughout the study, all slides being analyzed. 

### 3.4. Micronucleus Test

The micronucleus assay comprises a widely used test for the *in vivo* detection of clastogenic (which break up chromossomes) and aneugenic (which induce aneuploidy or abnormal chromossomic segregation) agents. The capacity to identify cells with a chromossomic defect bestows upon the micronucleus the property of being used as a biological marker to carcinogen exposure. The advantage of this test is its capacity of checking, *in vivo*, the state of a tissue exposed to a specific chemical agent, a hard-to-reproduce condition *in vitro*. This test is internationally accepted as part of the test battery recommended for the evaluation of mutagenic potential which are placed yearly in the world market. It was developed firstly in mouse bone marrow erythrocytes, but likewise in rats [40]. Preparations for the study of micronuclei frequency in bone marrow cells followed certain criteria recommended by Ribeiro *et al.* [41].

The animals were sacrificed thirty-six hours following the start of treatment with the tested agents; the skin was cut on the leg, and the femur was excised. The femur final proximal extremity was cut and the bone marrow channel being exposed. Bovine fetal serum was injected in the femur opening, and marrow cells placed in a previously-identified tube with each animal’s code. The cell suspension was centrifuged for 5 min, at 1,000 rpm, and the supernatant was discarded. Cell viability evaluation was performed with trypan blue following centrifuging, so that all samples would have the same cell concentration at the moment of smear preparation (1 × 10^6^ cells/mL). 

Smear preparation and slide dyeing a drop of cell suspension was placed onto a slide previously identified with each animal’s code, smearing being performed by means of another slide, two slides being made per animal. Slides were left at room temperature to dry, being later fixed in methanol for 10 minutes, and left at room temperature for drying. 

Slide dyeing was carried out with the Giemsa dye diluted in PBS (1:10), for 15 min. Following dyeing, slides were washed with distilled water for excess dye removal, being left to dry once more at room temperature. 

Cells were analyzed by 1000× magnification (immersion objective). Two-thousand polychromatic erythrocytes (PCE’s) from each animal (1,000 on each slide) were analyzed. 

### 3.5. Statistical Analysis

To evaluate the toxic effects of testing agents, data were analyzed using the ANOVA and Tukey–Kramer multiple comparison tests by statistical program InStat version 3.01 (GraphPad Software, San Diego, CA, USA). Significance level (*p* < 0.05) was adopted to compare data within the same experiment, where 6 animals per group were used. As recommended by Aguila *et al.* [42], five animals per group feature the minimum sample in quantitative studies and the experiment data could be conclusive. 

## 4. Conclusion

Despite the lack of genotoxic evidence, in the present experimental conditions, further studies should be ongoing, in order to evaluate these scintigraphy kits safety in patient diagnosis and treatment. The presented findings are important to the practice of the nuclear medicine procedures. 
